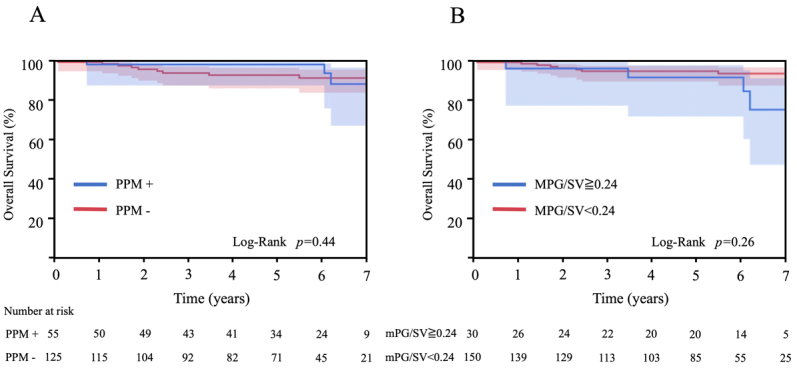# Clinical Impact of Flow-Adjusted Transprosthetic Pressure Gradient After Aortic Valve Replacement

**DOI:** 10.1016/j.atssr.2024.07.008

**Published:** 2024-07-26

**Authors:** Takahiro Ohmori, Arudo Hiraoka, Toshinori Totsugawa, Satoru Kishimoto, Yuki Yoshioka, Genta Chikazawa, Taichi Sakaguchi

**Affiliations:** 1 Department of Cardiovascular Surgery, The Sakakibara Heart Institute of Okayama, Okayama, Japan

## Abstract

**Background:**

It is still controversial whether prosthesis-patient mismatch (PPM) adversely affects long-term outcomes after aortic valve replacement (AVR). The aim of this study was to examine whether flow-adjusted pressure gradient is a valid new indicator of long-term outcomes.

**Methods:**

Data collected from 184 patients undergoing isolated AVR for severe aortic stenosis from October 2012 to September 2016 were analyzed. Flow-adjusted pressure gradient was defined as mean pressure gradient divided by stroke volume (MPG/SV). The effect of PPM and MPG/SV on long-term cardiac events and survival was evaluated.

**Results:**

Overall mortality was 8.2%, and the incidence of cardiac events was 9.2% (median follow-up period, 5.5 years). Moderate to severe PPM was present in 30.0% of patients and did not correlate with cardiac events (*P* = .13). The mean pressure gradient and stroke volume were also not predictive, but MPG/SV was significantly associated with cardiac events (*P* = 0.016), and the cutoff value of MPG/SV was 0.24 mm Hg/mL. MPG/SV ≥ 0.24 mm Hg/mL was detected as an independent risk factor (adjusted hazard ratio, 5.67; *P* < .001). The 5-year cardiac event-free rate was lower in patients with MPG/SV ≥ 0.24 mm Hg/mL (72.7% ± 9.6% vs 96.6% ± 1.7%; *P* < .001). Additionally, the left ventricular mass index at 1 month was significantly lower in patients with MPG/SV ≥ 0.24 mm Hg/mL (*P* = .028), although there was no significant difference at 6 months (*P* = .12).

**Conclusions:**

Flow-adjusted pressure gradient has the potential to be a better predictor of long-term outcomes after AVR.

## Visual Abstract

**Figure:**
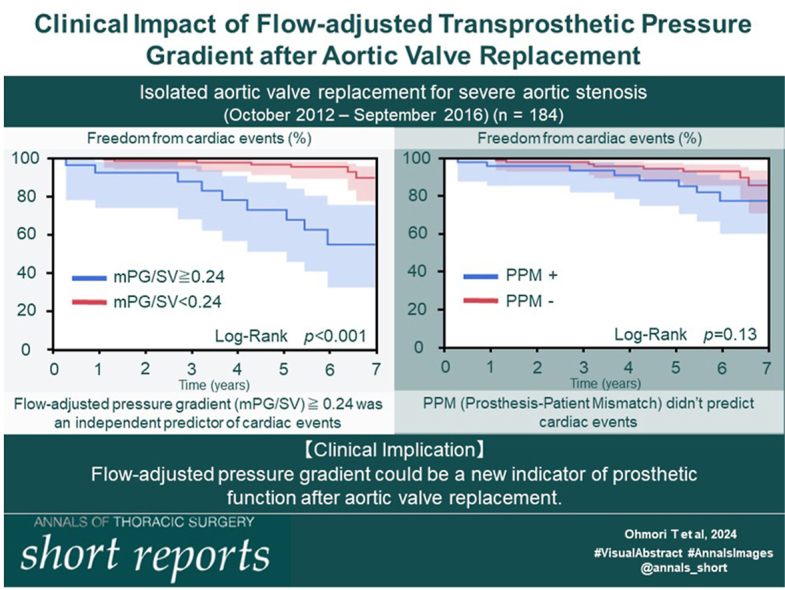

**In Short**

▪ Because greater flow means an increase of cardiac output, high gradient status should first be evaluated after excluding the influence of transprosthetic flow.
▪ Flow-adjusted transprosthetic pressure gradient was significantly associated with cardiac events after isolated AVR, whereas other conventional measures were not.
▪ A better early regression of the LV mass was obtained in patients with flow-adjusted low gradient status (<0.24 mm Hg/mL).

To decide the optimal treatment strategies for patients with aortic stenosis (AS) and evaluate the hemodynamic status after aortic valve replacement (AVR), avoidance of prosthesis-patient mismatch (PPM) is emphasized.^1^, ^2^, ^3^ However, many studies have failed to find a significant relationship between PPM and postoperative outcomes.^4^^,^^5^ Conversely, a mean pressure gradient (MPG) has been suggested as a valid indicator of prosthetic function and structural valve deterioration. However, the transprosthetic pressure gradient increases in proportion to transprosthetic flow. Given that greater flow (stroke volume [SV]) means an increase in cardiac output, adverse impacts of prosthetic valve status with high gradient should be evaluated first, after excluding the influence of transprosthetic flow.^6^ Previously, we reported that the flow-adjusted gradient has a potential to be a more sensitive indicator of functional mitral stenosis associated with clinical outcomes after mitral repair.^7^ On this foundation, the aim of this study was to evaluate the clinical impact of flow-adjusted MPG of the aortic prosthetic valve on long-term outcomes after isolated AVR for AS.

## Patients and Methods

### Patient Population

We retrospectively analyzed data from consecutive patients who underwent isolated AVR with a prosthetic valve for severe AS at the Sakakibara Heart Institute of Okayama, Japan, from October 2012 to September 2016. Patients who had undergone previous cardiac surgery (eg, concomitant coronary artery bypass grafting, mitral valve surgery, or ascending aorta replacement) were excluded. The remaining 184 patients were enrolled. This study was approved by the Institutional Ethics Committee in accordance with the ethical standards laid down in the 1964 Declaration of Helsinki and its later amendments (B201911-02, November 2, 2022). All operative clinical and echocardiographic data were collected from medical records. Consent from all patients for use of their medical records was obtained.

### Follow-up

The median follow-up period was 5.6 years (maximum, 9.5 years). The patients were followed up at 1 and 6 months after surgery and every year thereafter. The rate of follow-up at 1 month after surgery was 97.8% (180 of 184). The median time between surgery and the last echocardiogram was 5.4 years. The primary outcome comprised cardiac events, which consisted of cardiac death, hospital readmission for heart failure or arrhythmia, and reoperation for structural valve deterioration. The secondary outcome was a reduction in left ventricular mass index (LVMi) at 1 month. LVMi was calculated before and 1 month after surgery, by using the formula described by Devereux and coauthors.^8^

### Definition of Prosthesis-Patient Mismatch and Flow-adjusted Pressure Gradient

SV was measured from the left ventricular (LV) outflow tract area multiplied by the LV outflow tract velocity time integral by the pulsed-wave Doppler method. PPM was defined using the value of the effective orifice area index (EOAi), as described in previous reports: severe PPM if the EOAi was ≤ 0.65 cm^2^/m^2^, moderate PPM if the EOAi was >0.65 and ≤ 0.85 cm^2^/m^2^, and no PPM if the EOAi was >0.85 cm^2^/m^2^. Flow-adjusted pressure gradient was defined by MPG divided by LV SV (MPG/SV).

### Statistical Analysis

Continuous data are presented as mean ± SD. Categoric variables are given as the count and percentage of patients. Continuous data were compared with a Mann-Whitney *U* test. Categoric variables were compared using the χ^2^ test. When any expected frequency was less than 1, or if 20% of expected frequencies were less than or equal to 5, the Fisher exact test was used. MPG/SV threshold, which optimally predicted cardiac events, was determined using the Youden Index of the receiver operating characteristic curves. Risk factors for cardiac events were evaluated by multivariable Cox proportional hazard regression by using variables, with *P* values less than .05 in univariate analysis. The rate of cardiac events and survival after AVR was compared by the Kaplan-Meier model and the log-rank test. All data were analyzed using SAS software JMP version 12.0 (SAS Institute Inc).

## Results

### Preoperative and Operative Data

The mean age of patients was 75.5 years, and 61.4% were female, with a mean body surface area of 1.52 ± 0.17 m^2^. Patients’ characteristics and preoperative echocardiographic data are shown in Supplemental Table 1. Bioprosthetic and mechanical valves were implanted in 170 patients (92.4%) and 14 patients (7.6%), respectively. The type and size of implanted prostheses are shown in Supplemental Table 2.

### Postoperative Echocardiographic Data

The mean EOAi was 0.98 ± 0.21 cm^2^/m^2^. Severe and moderate PPM was observed in 4 (2.2%) and 50 patients (27.8%), respectively. The mean of MPG and MPG/SV was 11.6 ± 5.1 mm Hg and 0.19 ± 0.08 mm Hg/mL, respectively. The LV mass regression from baseline to 1 month was 18.0% ± 16.9%. Postoperative echocardiographic data are shown in Table 1.

**Table 1:** Postoperative Echocardiographic Characteristics

Variables	Values N = 180
Moderate or severe PVL	0 (0)
Moderate or severe MR	2 (1.1)
LVMi, g/m^2^	108.2 ± 29.6
LVMi regression, %	18.0 ± 16.9
Peak PG, mm Hg	22.9 ± 8.7
Mean PG, mm Hg	11.6 ± 5.1
EOA (equation of continuity), cm^2^	1.47 ± 0.32
EOAi, cm^2^/m^2^	0.98 ± 0.21
LVSV, mL	64.4 ± 13.6
Prosthesis-patient mismatch	54 (30.0)
Moderate	50 (27.8)
Severe	4 (2.2)
MPG/SV, mm Hg/mL	0.19 ± 0.08

Values are n (%) or mean ± SD.

EOA, effective orifice area; EOAi, effective orifice area index; LVMi, left ventricular mass index; LVSV, left ventricular stroke volume; MPG/SV, mean pressure gradient divided by stroke volume; MR, mitral regurgitation; PG, pressure gradient; PVL, paravalvular leakage.

### Overall Survival and Cardiac Events

**Figure 1:**
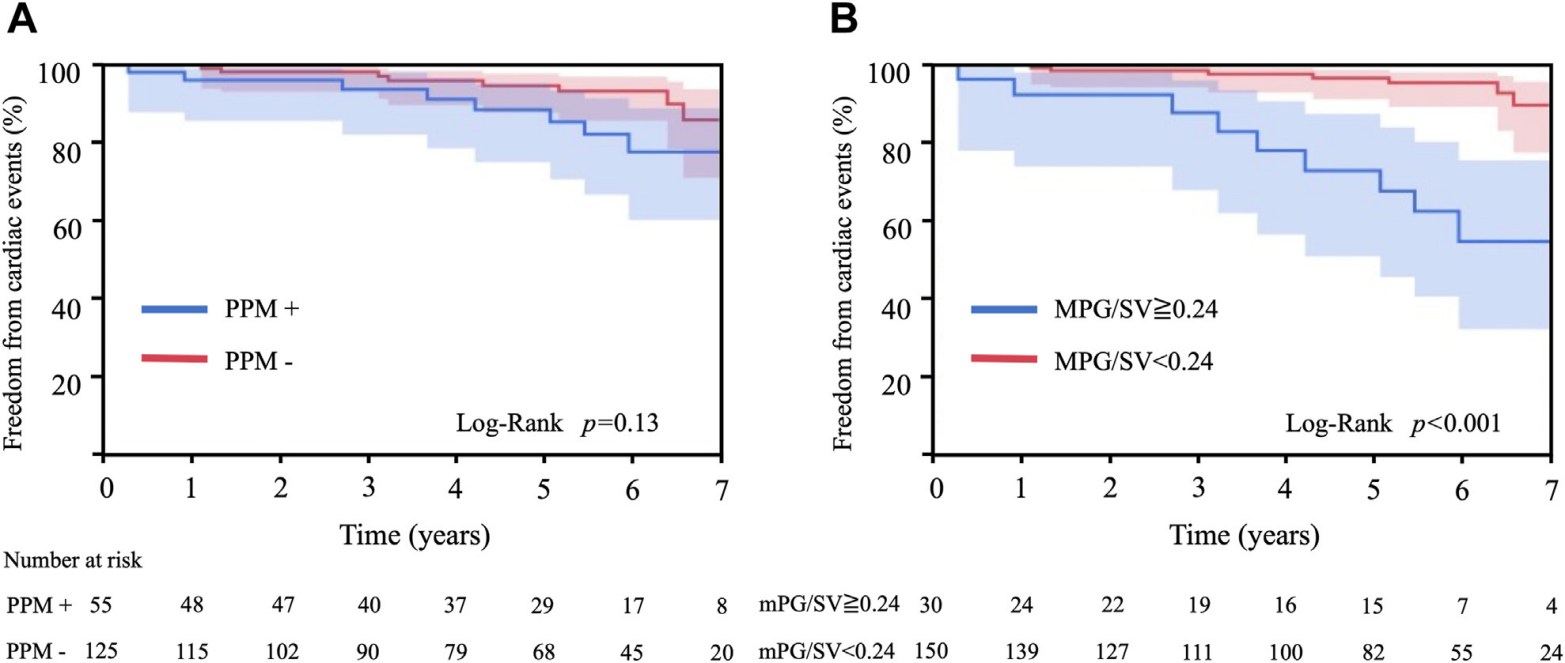
Kaplan-Meier curve for cardiac events stratified by (A) prosthesis-patient mismatch (PPM) and (B) mean pressure gradient divided by stroke volume (MPG/SV).

One patient died during the first month after AVR of intestinal ischemia, and 15 patients (8.2%) died during the follow-up periods. Cardiac events occurred in 17 patients (9.2%) (Supplemental Table 3). The overall cardiac event-free rate at 5 years was 92.7%. In the univariate analysis for cardiac events of 180 patients who had echocardiograms 1 month after AVR, moderate to severe PPM was not associated with cardiac events, and there was no significant difference in the 5-year event-free rate between patients with and without moderate to severe PPM (88.5% ± 4.9% vs 94.7% ± 2.4%; *P* = .13) (Figure 1A). Neither MPG (*P* = .13) nor SV (*P* = .9) had a significant correlation with cardiac events. There was a significant correlation in MPG/SV (per 0.1) and LV mass regression (per 10%) with cardiac events (*P* = .016 and .018). By the receiver operating characteristic analysis, the cutoff value of MPG/SV as a predictor of cardiac events was 0.24 (area under the curve = 0.66; *P* = .015). Sensitivity was 52.9%, and specificity was 87.2%. On the basis of the cutoff value, the patients were divided into 2 groups: patients with a high gradient (MPG/SV ≥ 0.24 mm Hg/mL) and patients with a low gradient (MPG/SV < 0.24 mm Hg/mL). Thirty patients (16.7%) were included in the high-gradient group. There was a significant difference in the 5-year event-free rate between the groups (72.7% ± 9.6% vs 96.6% ± 1.7%; *P* < .001) (Figure 1B). There was no significant difference in the survival rate at 5 years after AVR between the high- and low-gradient groups (91.6% ± 5.7% vs 94.8% ± 1.9%; *P* = .26), as well as the groups with and without PPM (98.0% ± 1.9% vs 92.6% ± 2.5%; *P* = .44) (Supplemental Figure).

By multivariable analysis, MPG/SV ≥ 0.24 was detected as an independent predictor of risk factors of cardiac events (hazard ratio, 5.67; 95% CI, 2.07-15.6; *P* < .001) (Table 2).

**Table 2:** Predictors of Cardiac Events After Aortic Valve Replacement for Aortic Stenosis at Univariable and Multivariable Analysis

Variables	Univariable			Multivariable		
	HR	95% CI	*P* Value	Adjusted HR	95% CI	*P* Value
Preoperative data						
Age, per 1 y	1.04	0.98-1.11	.21	...	...	...
Female	1.12	0.41-3.03	.82	...	...	...
BMI, per 1 kg/m^2^	1.06	0.94-1.19	.30	...	...	...
NYHA functional class Ⅲ-Ⅳ	0.78	0.18-3.41	.74	...	...	...
LVEF, per 1%	0.99	0.95-1.05	.80	...	...	...
Serum creatinine, per 1 mg/dL	1.12	0.89-1.31	.21	...	...	...
Moderate or severe AR	2.41	0.85-6.85	.10	...	...	...
LVMi, per 10 g/m^2^	1.08	0.95-1.21	.21	...	...	...
Postoperative data						
Moderate to severe PPM	2.07	0.80-5.37	.13	...	...	...
MPG, per 1 mm Hg	1.05	0.97-1.11	.13	...	...	...
LVSV, per 10 mL	0.98	0.68-1.38	.90	...	...	...
MPG/SV, per 0.1	1.58	1.04-2.21	.016^a^	...	...	...
MPG/SV ≥ 0.24 mm Hg/mL	7.28	2.80-19.0	<.001^a^	5.67	2.07-15.6	<.001^a^
LV mass regression, per 10%	0.71	0.53-0.94	.018^a^	0.79	0.61-1.04	.09

AR, aortic regurgitation; BMI, body mass index; HR, hazard ratio; LV, left ventricular; LVEF, left ventricular ejection fraction; LVMi, left ventricular mass index; LVSV, left ventricular stroke volume; MPG, mean pressure gradient; MPG/SV, mean pressure gradient divided by stroke volume; NYHA, New York Heart Association; PPM, prosthesis-patients mismatch.

a Significance is *P* < .05.

### Left Ventricular Mass Regression

**Figure 2:**
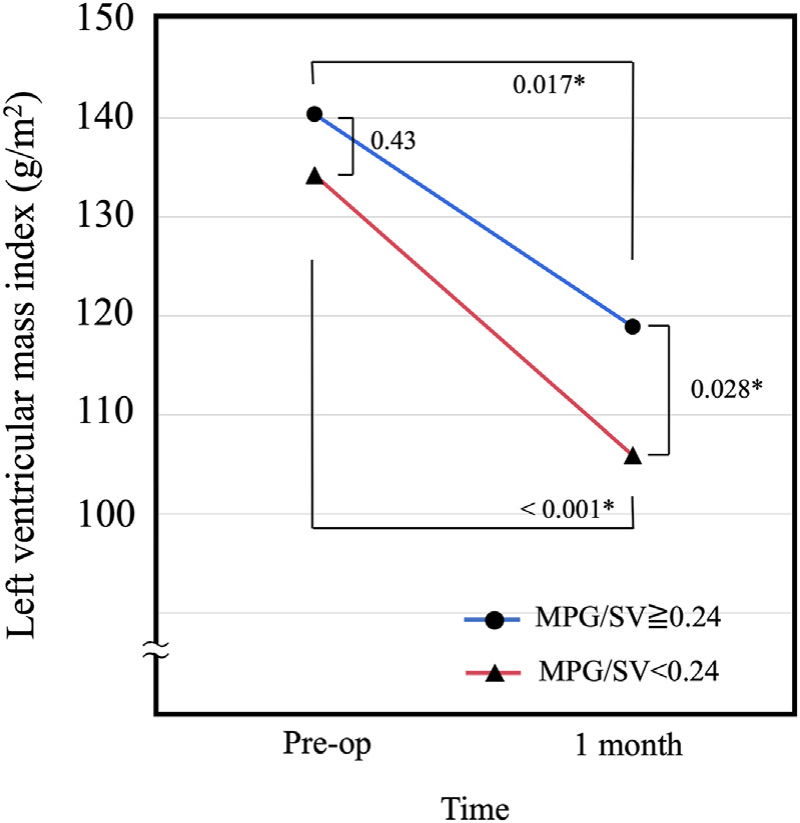
Left ventricular mass index before (Pre-op) and 1 month after aortic valve replacement according to mean pressure gradient divided by stroke volume (MPG/SV) ≥ 0.24 and < 0.24 mm Hg/mL. ∗There was a significant difference in change of left ventricular mass index between the two groups at one month (*P* = .028).

There was no significant difference in the baseline LVMi between the low- and high-gradient groups (*P* = .43). LVMi significantly decreased at 1 month in both groups (high-gradient group, *P* = .017; low-gradient group, *P* < .001). The low-gradient group showed a significantly lower LVMi than the high-gradient group at 1 month (*P* = .028) (Figure 2), although there was no significant difference in LVMi between both groups at 6 months (*P* = .12).

## Comment

The main findings of this study are as follows:

1. Flow-adjusted MPG (MPG/SV) was an independent risk factor for cardiac events, whereas moderate to severe PPM, MPG, and SV were not. The cutoff value of MPG/SV as a predictor of cardiac events was 0.24 mm Hg/mL.
2. Neither moderate to severe PPM nor flow-adjusted MPG was associated with all-cause mortality.
3. Better LV mass regression was observed in patients with a low gradient (MPG/SV <0.24 mm Hg/mL).

SV is known to decrease transiently after surgical AVR and then increase over time. This is why the relationship between early postoperative low flow and late outcomes is still unclear, although preoperative low flow is considered a poor prognostic factor.^9^ Conversely, the MPG is widely used as a variable to assess the hemodynamic function of a prosthetic valve. A high transprosthetic pressure gradient leads to insufficient reduction of the LV burden. However, Koene and colleagues^10^ showed that the transprosthetic MPG did not predict long-term survival. This is partly because the pressure gradient varies in proportion to the transprosthetic flow. Patients with high flow have an apparently high-pressure gradient. Thus, both measures are inadequate as indicators of late outcomes after AVR. In contrast, MPG/SV is a value relatively independent of the circulatory condition as a result of adjustment by flow. It reflects more accurately the resistance generated by the prosthetic valve and is considered useful as an indicator for assessing prosthetic valve function. It is a valid variable for stratifying prognosis after AVR in the long term, and patients classified as having a poor prognosis may need careful pharmacologic therapy to inhibit LV remodeling.

### Study Limitations

First, this was a single-center retrospective study. Second, because this cohort included physically small Asian patients, the prevalence of PPM may be different in another population.

### Conclusion

Flow-adjusted pressure gradient has potential to be a better predictor of long-term outcomes after AVR compared with moderate to severe PPM or MPG. A better early regression of the LV mass was obtained in patients with flow-adjusted low gradient status (MPG/SV <0.24 mm Hg/mL).

## Figures and Tables

**Supplementary Figure:**