# Supplementary material for: Clinical Impact of Flow-Adjusted Transprosthetic Pressure Gradient After Aortic Valve Replacement
Source: Ann Thorac Surg Short Rep. 2024 Jul 26;3(1):42–6. doi: 10.1016/j.atssr.2024.07.008 (PMC11910783; doi:10.1016/j.atssr.2024.07.008)
Supplement: Supplementary Figure 1 and Supplementary Tables 1-3 [file mmc1.docx]

**Figure Legends**

**Supplemental Figure.** Kaplan-Meier curve for all-cause mortality stratified by PPM (A) and MPG/SV (B). PPM, prosthesis-patient mismatch: MPG/SV, mean pressure gradient divided by stroke volume.

**Supplemental Table 1. Baseline patients’ characteristics**

| **Variable** | **(n = 184)** |
| --- | --- |
| **Clinical characteristics** |  |
| **Age, years** | 75.5 ± 8.8 |
| **Female** | 113 (61.4%) |
| **BSA, m^2^** | 1.52 ± 0.17 |
| **BMI, kg/m^2^** | 23.0 ± 4.0 |
| **NYHA class** |  |
| **Ⅰ** | 53 (28.8%) |
| **Ⅱ** | 95 (51.6%) |
| **Ⅲ** | 28 (15.2%) |
| **Ⅳ** | 8 (4.3%) |
| **Serum creatinine, mg/dL** | 1.52 ± 2.19 |
| **BNP, pg/mL** | 318.8 ± 515.6 |
| **Dialysis** | 18 (9.8%) |
| **Emergent / Urgent operation** | 8 (4.3%) |
| **Echocardiographic characteristics** |  |
| **LV ejection fraction, %** | 65.5 ± 10.6 |
| **LV fractional shortening, %** | 36.7 ± 7.9 |
| **LV end-diastolic diameter, mm** | 45.1 ± 5.9 |
| **LV end-systolic diameter, mm** | 29.0 ± 6.4 |
| **LV posterior wall dimension, mm** | 12.0 ± 1.9 |
| **LV septal wall dimension, mm** | 12.2 ± 2.0 |
| **LVM, g** | 206.0 ± 63.9 |
| **LVMi, g/m^2^** | 135.9 ± 39.0 |
| **Vmax, m/s** | 4.7 ± 0.8 |
| **peak PG, mmHg** | 91.0 ± 30.2 |
| **mean PG, mmHg** | 52.9 ± 18.1 |
| **AVA (equation of continuity), cm^2^** | 0.73 ± 0.17 |
| **AVAi, cm^2^/m^2^** | 0.48 ± 0.11 |
| **moderate or severe AR** | 33 (17.9%) |
| **moderate or severe MR** | 3 (1.6%) |
| **moderate or severe TR** | 3 (1.6%) |
| **TRPG, mmHg** | 25.7 ± 8.6 |
| **e/e’** | 19.3 ± 8.5 |

BSA, body surface area; BMI, body mass index; NYHA, New York Heart Association; BNP, brain natriuretic peptide; LV, left ventricular; LVM, left ventricular mass; LVMi, left ventricular mass index; PG, pressure gradient; AVA, aortic valve area; AVAi, aortic valve area index; AR, aortic regurgitation; MR, mitral regurgitation; TR, tricuspid regurgitation; TRPG, tricuspid regurgitation pressure gradient

**Supplemental Table 2. Types and sizes of prosthetic valves**

| **Prosthesis Type** | **Size (mm)** | | | | | | | | | | **Total** |
| --- | --- | --- | --- | --- | --- | --- | --- | --- | --- | --- | --- |
|  | **16** | **17** | **18** | **19** | **20** | **21** | **22** | **23** | **25** | **27** |  |
| **Bioprosthetic valves** |  |  |  |  |  |  |  |  |  |  | 170 |
| **Carpentier-Edwards Perimount Magna** |  |  |  | 35 |  | 48 |  | 34 | 15 | 3 | 135 |
| **Mosaic** |  |  |  | 6 |  | 11 |  | 2 |  |  | 19 |
| **Trifecta GT** |  |  |  | 6 |  | 5 |  |  |  |  | 11 |
| **Mitroflow** |  |  |  | 4 |  |  |  |  |  |  | 4 |
| **Crown PRT** |  |  |  | 1 |  |  |  |  |  |  | 1 |
| **Mechanical valves** |  |  |  |  |  |  |  |  |  |  | 14 |
| **ATS AP** | 1 |  | 1 |  | 4 |  | 4 |  |  |  | 10 |
| **St. Jude Medical Regent** |  | 3 |  | 1 |  |  |  |  |  |  | 4 |

**Supplemental Table 3. Postoperative date**

| **Variables** | **(n = 184)** |
| --- | --- |
| **30-day mortality** | 1 (0.5%) |
| **All cause death** | 15 (8.2%) |
| **Heart failure** | 2 (1.1%) |
| **Cerebral hemorrhage** | 3 (1.6%) |
| **Cancer** | 2 (1.1%) |
| **Infection** | 2 (1.1%) |
| **Unknown** | 4 (2.2%) |
| **Others** | 2 (1.1%) |
| **Cardiac events** | 17 (9.2%) |
| **Heart failure** | 6 (3.3%) |
| **Arrhythmia** | 7 (3.8%) |
| **Structural valve deterioration** | 4 (2.2%) |
